# The Effects of Sleep Extension on Sleep, Performance, Immunity and Physical Stress in Rugby Players

**DOI:** 10.3390/sports6020042

**Published:** 2018-05-10

**Authors:** Richard Swinbourne, Joanna Miller, Daniel Smart, Deborah K. Dulson, Nicholas Gill

**Affiliations:** 1 Faculty of Sport and Recreation, Auckland University of Technology, Auckland 0627, New Zealand; deborah.dulson@aut.ac.nz; 2 Nutrition Department, Singapore Sport Institute, Singapore 397630, Singapore; 3 Physiology Department, Australian Institute of Sport, Canberra 2617, Australia; Jo.Miller@ausport.gov.au; 4 Sports Research Institute New Zealand, Auckland University of Technology, Auckland 0627, New Zealand; dan.smart@nzrugby.co.nz; 5 Faculty of Health, Sport and Human Performance, University of Waikato, Hamilton 3240, New Zealand; nicholas.gill@nzrugby.co.nz

**Keywords:** sleep extension, collision sports, physical stress, recovery, performance

## Abstract

(1) Background: The purpose of the present study was to examine the efficacy of sleep extension in professional rugby players. The aims were to: (i) characterise sleep quantity in elite rugby players and determine changes in immune function and stress hormone secretion during a pre-season training programme; (ii) evaluate the efficacy of a sleep extension intervention in improving sleep, markers of physical stress, immune function and performance. (2) Methods: Twenty five highly trained athletes from a professional rugby team (age (mean ± SD) 25 ± 2.7 years; height 1.87 ± 0.07 m; weight 105 ± 12.1 kg) participated in a six week pre-post control-trial intervention study. Variables of sleep, immune function, sympathetic nervous activity, physiological stress and reaction times were measured. (3) Results: Sleep extension resulted in a moderate improvement in sleep quality scores ([mean; ± 90% confidence limits] −24.8%; ± 54.1%) and small to moderate increases in total sleep time (6.3%; ± 6.3%) and time in bed (7.3%; ± 3.6%). In addition, a small decrease in cortisol (−18.7%; ± 26.4%) and mean reaction times (−4.3%; ± 3.1%) was observed following the intervention, compared to the control. (4) Conclusions: Professional rugby players are at risk of poor sleep during pre-season training, with concomitant rises in physical stress. Implementing a sleep extension programme among professional athletes is recommended to improve sleep, with beneficial changes in stress hormone expression and reaction time performance.

## 1. Introduction

Adequate sleep is considered critical for optimal performance and has been recognised as the most important recovery modality by a large number of elite team sport athletes, including rugby union [1]. Rugby union is a repetitive high intensity collision sport where the athlete’s ability to train at high intensity to maintain or improve physical qualities such as speed, power and strength and to optimise recovery is vital. This is particularly relevant during the pre-season training phase where the athlete is placed under a high degree of exercise stress to induce adaptations in physical fitness. It would appear that adequate amounts of sleep quality and quantity allow these specific adaptations to occur [2]. Conversely, poor sleep has been linked with an increased incidence of fatigue related injury [3], hormonal and metabolic disturbances [4,5] and elevated sympathetic nervous system activity [6].

Whilst the importance for athletes to achieve adequate sleep is widely accepted to enable athletic potential, it appears that athletes are at risk of poor sleep [7,8,9,10,11]. Moreover, sleep extension appears to impact positively on athlete wellbeing [12] and performance [10].

Furthering value to the strategy of sleep extension, sleep perturbations can alter immune and inflammatory status [13]. Associations have been observed between abnormalities of immune function and various forms of sleep disruption [14] as well as increased training load in rugby players [15].

Sleep and nervous system recovery also appear to be intricately entwined [6]. Additionally, salivary alpha amylase (sAA) is a marker of autonomic nervous system (ANS) stress [16]. However, the application of sAA as a surrogate marker of ANS stress in relation to sleep in athletes appears to be novel. Cortisol is also an identified indicator of endocrine response to competitive high intensity combative sports [17]. Furthermore, sleep restriction has been shown to cause increases in cortisol concentrations [6]. Considering that cortisol is the primary catabolic hormone, increasing protein degradation and decreasing protein synthesis [18], reducing elevated cortisol may be an important consideration for elite rugby players.

It is important to investigate the effect sleep has on athlete health and performance. Understanding the impact sleep extension may have on athletes should involve indices of sleep quality, physical performance, immune function and research related stress using quality research methodology. Therefore, the purpose of the present study was to examine the efficacy of sleep extension during a professional rugby pre-season training programme. Specifically the aims were (i) to characterise sleep quantity obtained by elite rugby players and determine changes that may occur in immune function and stress hormone secretion during a pre-season training programme; (ii) evaluate the efficacy of sleep extension during pre-season training and resultant improvements in sleep quantity and quality and markers of physical stress, immune function and performance.

## 2. Materials and Methods

### 2.1. Participants

Twenty five highly trained male rugby union athletes volunteered to participate in the present study (Table 1). Participants provided written informed consent prior to participation. All participants were in good health and none had previously been diagnosed with a sleeping disorder. Participants were permitted to consume caffeine, alcohol and training supplements during the course of the study. The study was approved by the Auckland University of Technology Ethics Committee (Ethics application number 13/373).

### 2.2. Research Design

A pre-post control-trial intervention study design was implemented to collect data on sleep quantity and quality, salivary markers of immune function, sympathetic nervous activity and physiological stress and reaction times during two three-week pre-season training phases. Participant’s completed an initial three-week high intensity training block (control), followed by a two week maintenance period. A sleep extension intervention was then performed during a second three-week high intensity training block. Qualitative sleep data was collected weekly, and saliva samples were collected at the beginning and end of the control and sleep extension intervention. Reaction times were assessed at baseline during the control, and at the beginning and end of the sleep extension intervention. During the intervention, participants were instructed to try and achieve ten hours sleep per night and educated about how to obtain more sleep via sleep scheduling advice and strategic napping, as well as controlling sleep environment factors such as light, noise and temperature. Training programmes were designed by the team’s strength and conditioning coach with both blocks comparable in volume and intensity. Participants trained up to three times per day consisting of either weight training or high intensity running.

### 2.3. Sleep

#### 2.3.1. Qualitative Sleep and Daytime Sleepiness Assessments

Participants self-perceived sleep quality was assessed using the Pittsburgh Sleep Quality Index (PSQI) questionnaire [19] and daytime sleepiness via the Epworth Sleepiness Scale (ESS) questionnaire [20]. Participants recorded sleep times and naps in sleep diaries which were completed while participants were wearing activity monitors (wGT3Xp, Actigraph, Pensacola, FL, USA). Questionnaires and sleep diaries were completed in the morning upon arrival at the training venue. Questionnaires were completed at the end of each training week during the control and sleep extension phase.

Pittsburgh Sleep Quality Index Questionnaire. The PSQI provides a subscale rating of subjective sleep quality, as well as sleep latency, sleep duration, and habitual sleep efficiency. Some components relate to sleep disturbances, use of sleeping medication, and daytime dysfunction. The sum of the scores for these seven components yields a global score with a range of 0–21 that is a composite of sleep quantity and quality [21]. A global PSQI score greater than five has resulted in a diagnostic sensitivity of 89.6% and specificity of 86.5% in distinguishing ‘good’ and ‘poor’ sleepers [19].

Epworth Sleepiness Scale Questionnaire. The ESS is a simple questionnaire measuring the general level of daytime sleepiness, or average sleep propensity experienced by an individual [22]. The questionnaire yields a global score with a range of 0–24. The ESS has been proven to be a valid and reliable measure of objective sleepiness [22] and a practical evaluative tool that has been applied to athletes [10,23]. The ESS may compliment the PSQI and provide information on whether an athlete is experiencing daytime functional disturbance from poor sleep quality, which may impact on cognition and training quality.

#### 2.3.2. Quantitative Sleep Assessment

##### Actigraphy

During the study all participants slept in their residential homes. Participants wore wrist watch activity monitors (wGT3Xp, Actigraph, Pensacola, FL, USA) on their non-dominant wrist, for two to five nights at a time. During the control phase fourteen participants wore activity monitors twice during both early and late stages of the training block. Due to participant drop out, 11 participants wore activity monitors twice during both early and late stages of the sleep extension intervention.

Individual nights of sleep were analysed for the following variables: Total time in bed (h:min), sleep latency (min), overnight sleep duration (h:min), total sleep time (h:min), sleep efficiency (%), wake after sleep onset (WASO) (min) and number of awakenings. Actigraphy has been validated as a tool to measure sleep patterns in healthy adults [24] and appears to be a practical and effective method to monitor athletes in their daily training environment.

##### Saliva Sampling

Saliva samples were collected on day four and the final day of the 18 day control phase and on day three and day 19 of the 19 day sleep extension intervention for analysis of salivary IgA, cortisol and α-amylase. Saliva was collected in the morning immediately upon arrival at the training venue. Approximately one hour prior to saliva collection, participants consumed a standardised high carbohydrate, moderate protein and low fat breakfast for the purposes of training quality. Dietary compliance was verbally checked by the researcher. Every attempt was made to keep the participants in their habitual pre-training routines, and to control morning activity and potential stressors at both collection time points. 

The saliva collection procedure has been previously described in detail [25]. Timed un-stimulated whole mixed saliva samples were collected over a two minute period, unless insufficient volume had been produced, in which case the collection period was increased to three minutes. Saliva samples were frozen at −20 °C until analysed.

Saliva volume was estimated by weighing the bijou tubes to the nearest milligram before and after saliva collection. Saliva density was assumed to be 1.00 g·mL^−1^ [26]. The saliva flow rate (μL·min^−1^) was determined by dividing the volume of saliva by the collection time. For analysis, samples were thawed and then spun at 13,400 rpm for 2 min, and the saliva was subsequently analyzed for s-IgA, cortisol and *α*-amylase using immunoassay and spectrophotometric methods. Both s-IgA and cortisol concentration were determined using commercially available ELISA kits (DRG SLV-4636 & SLV 2930, DRG International, Inc., Springfield, New Jersey, NJ, USA). The α-amylase activity was measured using a commercially available kit (Infinity TM Amylase Liquid Stable Reagent, Thermo Scientific, UK) [27]. s-IgA secretion rate was calculated by multiplying s-IgA concentration (mg·L^−1^) by saliva flow rate (μL·min^−1^) and dividing by 1000 to give s-IgA secretion rate (μg·min^−1^). The secretion rate of α-amylase (U·min^−1^) was calculated by multiplying the saliva flow rate by the α-amylase activity (U·mL^−1^). The intra-assay coefficient of variation was 3%, 3.3% and 1.7% for s-IgA, cortisol and α-amylase, respectively.

##### Reaction Times

Reaction times were tested at baseline during the control, and at the beginning and end of the sleep extension intervention. The five minute psychomotor vigilance test (PVT) (PVT-192, Ambulatory monitoring, Inc., New York, NY, USA) was selected to test participant reaction times. The PVT is a fully electronic, computerized test-presentation and data capture system for simple visual reaction time monitoring. The PVT is commonly used to assess the impact of sleep loss, sustained wakefulness and time of day on neuro-behavioural performance [28]. The five minute PVT is a valid and reasonable substitute for the ten minute PVT when used in the field, and there is a strong correlation in response time between the five and ten minute tests (r = 0.88, *p* < 0.001) [28]. Prior to each test, participants were given a standard explanation of test procedures and provided with a standardised 60 s sample visual reaction time trial familiarisation. Data was analysed for mean reaction time (ms), fastest and slowest 10% reaction times (ms) and total errors using React PVT software.

##### Statistical Analysis

All data were analysed using an Excel spreadsheet for analysis of pre-post controlled trials, which was set at 90% confidence limits [29]. All biochemical data were log-transformed prior to analysis to reduce non-uniformity of error. These data were back transformed and expressed as the parametric median, with errors expressed as 90% confidence limits. Standardised mean changes and differences between the trials changes were used to assess magnitudes of effects by dividing the appropriate between-player standard deviation. Standardised effects were defined as using a modified Cohen scale: <0.2 = trivial, 0.2–0.59 = small, 0.6–1.19 = moderate, 1.2–1.99 = large, >2.0 = very large [30]. The effect was deemed unclear if its confidence interval overlapped the thresholds for small positive and negative effects.

## 3. Results

### 3.1. Sleep Questionnaires

Control and sleep extension intervention values (mean ± SD) for subjective sleep quality (PSQI) and sleepiness (ESS) global scores are presented in Table 2. Overall, sleep extension resulted in a moderately larger improvement in sleep quality scores ([mean; ± 90% confidence limits] −24.8%; ±54.1%) compared to control.

#### 3.1.1. Actigraphy

Actigraphy sleep data was collected from 84 nights (control) and 74 nights (sleep extension) of activity monitor wear time. Measures of all sleep variables across both treatment periods are presented in Table 3. There were small and moderate differences between the control and sleep extension intervention’s changes in Total Sleep Time (6.3%; ±6.3%) and Total Time in Bed (7.3%; ±3.6%) respectively.

#### 3.1.2. Saliva Analysis

Mean salivary IgA and α-amylase secretion rate concentrations and cortisol concentrations are presented in Table 4. A small difference in cortisol changes between treatments was observed (−18.7%; ±26.4%).

#### 3.1.3. Reaction Times

Sleep extension reaction time variables are shown in Table 5. After sleep extension there was a small decrease in mean reaction times (−4.3%; ±3.1%) and fastest 10% time (3.3%; ±2.9%) and a moderate increase in the slowest 10% time (11.7%; ±7.2%).

## 4. Discussion

The purpose of the present study was to characterise sleep quantity in elite rugby players, determine changes in immune function and stress hormone secretion, and evaluate the efficacy of a sleep extension intervention during a pre-season training programme. Extending sleep resulted in a greater increase in time in bed, total sleep time and sleep quality, a decrease in physiological stress and improved reaction time performance compared to the control. However, sleep extension did not improve indices of daytime sleepiness, immune function or sympathetic nervous system activity.

Following the three-week sleep extension intervention, improvements in the rugby player’s total sleep times support previous findings [10,31]. Mah and colleagues [10] observed significant improvements in extended night time sleep (average nightly increase 110.9, ±79.7 min) and average sleep time (507.6, ±78.6 min), compared to baseline (average sleep time 400.7, ±61.8 min). Similarly, Famodu [31] found female track athletes significantly increased total sleep time by 22 min during a one week sleep extension period. Thus, athletes appear to respond well to education and encouragement to sleep more. However, there is a lack of agreement in the literature concerning the benefits of sleep extension.

Sleep extension studies in athletes have demonstrated improvements in sprint and reaction times, skill execution, overall ratings of physical and mental well-being and mood [10,12,31]. Furthermore, baseline ESS sleepiness scores (9.64 ± 3.8) have also been shown to improve in athletes following sleep extension (3.36 ± 1.69) [10]. Conversely, twenty minutes of sleep extension in Australian Football players did not significantly affect perceived or training stress or reaction times [12]. Similarly, one additional hour of sleep extension in female track athletes showed no significant effects on variables of power, fatigue or reaction times [31]. Considering the contradictory findings between the present study and aforementioned studies, results regarding a lack of improvement in sleepiness and skill execution, and beneficial changes in reaction times, an athlete’s response to sleep extension appears to be highly variable. Indeed, a successful response to sleep extension may depend on various factors, including individual sleep requirements, training load and intensity, the length and timing of sleep extension and the quality of habitual sleep prior to sleep extension.

With respect to habitual sleep among athletes prior to sleep extension, Mah and colleagues [10] also observed similarly poor sleep times measured by actigraphy as compared to the present study. Together, these data support previous findings that have characterised poor normative sleep behaviour in athletes [8,9,11,32,33,34]. These findings suggest that intensive efforts to improve habitual sleep behaviour in athletes are warranted. Furthermore, sleep times decreased as training progressed during the control phase of the present study. A similar finding has been observed in over-reached endurance athletes [35], suggesting that athletes are particularly prone to poor sleep during phases of hard training, further highlighting the importance of monitoring and educating athletes on sleep.

The duration and timing of sleep extension is a likely determinant of the benefits of a sleep extension intervention. Participants in the present study were set the same sleep target (10 h) as in-season basketball athletes [10], yet did not extend their average nightly sleep to the same degree. Similarly, an even smaller, and non-significant improvement in nightly sleep duration was observed (~10 min) amongst Australian Football players undertaking a six week sleep extension programme [12]. In the present study, comparatively less sleep extension may in part explain the lack of difference in sleepiness scores between the control and intervention phases. Furthermore, the rugby players may have experienced more disrupted sleep due to a higher training volume and intensity within a pre-season phase, hot environmental conditions, or were simply unable to sleep as much as college athletes. From results available, the three-week sleep extension period in the present study appears to be more beneficial than one week [31], but not as effective as 5–7 weeks of significantly extended sleep [10]. Nightly sleep duration achieved by an athlete is also an important consideration. More conservative sleep durations may require a longer period of sleep extension, thus allowing an athlete opportunity to more fully dissolve existing sleep debt. While sleep extension studies longer than two months are lacking, it would seem prudent to suggest that extending sleep time and improving sleep quality among athletes is a habitual long term consideration, rather than an acute strategy. In particular, an athlete’s habitual sleep duration may be a critical consideration to allow physical and neural adaptations adequate time to be realised, thus optimising the competitive advantages that may be available from sleep. 

The health and performance benefits available to athletes by reducing sleep debt seem considerable [10,36,37]. Among these benefits is the potential for sleep to enhance immune function and mitigate elevations in physical stress. Specifically, cortisol is a particularly relevant stress hormone to athletes due to the effect that high volume and intensity training has in elevating cortisol [38] and its negative influence over body composition [18]. Furthermore, there is an emerging concept that cortisol has a dose response effect with training, and may, together with testosterone, regulate long term changes in muscle growth and performance, especially with resistance training [18]. In light of the potential benefits of suppressing cortisol during pre-season training, the beneficial effect that sleep extension had on mitigating a rise in cortisol may provide a platform to support enhanced adaptive outcomes.

There was a lack of clear change in markers of immune function and sympathetic nervous activity between the control and intervention in the present study. However, sleep and immune function are intricately linked [39]. Upper respiratory tract infection has been associated with changes in s-IgA and alterations in training load in elite rugby union players [15]. This may contribute to reductions in mucosal immunity, which, when lowered, predispose rugby players to an increased risk of illness [15]. Results have consistently shown that sAA, as a marker of ANS activity, changes in response to psychological stress [16]. Sleep deprivation, as a source of stress, is a known stimulant of sympathetic nervous system activity [6]. Whilst there is strong scientific rationale for sleep extension, and parallel reductions in cortisol, to improve markers of immune function and sympathetic nervous activity in athletes, results from the present study do not support this hypothesis. Saliva samples were only taken twice, at baseline and end of treatment. Sampling frequency may have affected results due to the possibility of a high degree of individual variability in biochemical markers. More frequent sampling of salivary bio markers in future sleep extension studies, with more long term sleep extension periods, is warranted to better explore the effect improved sleep might have on an athlete’s immunity and ANS activity.

A further aim of the study was to evaluate the efficacy of a sleep extension intervention in improving athlete performance and specifically reaction times. The small beneficial decrease in reaction times after sleep extension contrast with the lack of improvement observed by Van Ryswyk et al. [12], but do agree with findings by Mah and colleagues [10], reflecting a positive change in athlete performance. Such a change in performance may be explained by increased sleep positively influencing neural functioning. Baseline reaction times were faster in the present study than those reported by Mah et al., [10], possibly due to the elite professional athletes in the present study possessing more efficient nervous system functioning. Thus, the significant reaction time improvements that were still achieved in the present study point towards a distinct competitive advantage that may be obtained from improved sleep within an elite sporting environment.

The improvement of poor sleep in elite collision sport athletes appears to confer worthwhile benefits regarding the absorption of physiological stress and enhancing performance during intensive training, suggesting several practical applications for collision sport environments. Sleep extension education and sleep related behaviour change during intensive training phases is strongly recommended in conjunction with sleep monitoring. Education is appropriate at a group level, but ensuring adequate time with individual athletes, even in busy elite programmes, should be prioritised for optimal learning and outcomes. Athletes appear to respond well to seeing their individual sleep data, and use this as an incentive to improve habits. Whilst educating athletes in the area of sleep hygiene and sleep extension is important, empowering them with opportunities to sleep via appropriately planned training programmes is a prudent consideration. Factors such as family and social circumstances, work, study and commute times to trainings in large cities, as well as early and late training times may all disrupt sleep opportunities. Training times are often a controllable factor for coaches to be mindful of. Indeed coaches may amplify training adaptations by prioritising sleep when scheduling training. Sleep may be considered an anabolic and restorative process, allowing an athlete to mitigate physiological stressors. An alternative way to consider this point is that a well slept athlete may tolerate more training stress, possibly resulting in greater adaptation. Neurocognitive performance improvements are also possible during intense training phases, and extending sleep extension into competition phases may be beneficial for team sports where neurocognitive performance is an essential element of sporting success.

## 5. Conclusions

In summary, a sleep extension intervention during a professional rugby pre-season training programme resulted in athletes improving total sleep time and quality, with beneficial changes in stress hormone expression and reaction time performance compared to a control. The amelioration of the cortisol response to intense pre-season training, and enhanced neurological functioning, may be attributed to improvements in indices of sleep and/or dissolving sleep debt. Sleep extension did not improve other variables of sleep quality or daytime sleepiness. Further sleep extension research is therefore required among elite rugby players, involving comprehensive indices of sleep quality, physical health, recovery and performance over longer term periods. The results of the present study suggest that sleep education and promoting sleep extension among elite athletes is a worthwhile and effective measure in the pursuit of optimal performance.

## Figures and Tables

**Table 1:** Descriptive statistics of professional rugby union players (n = 25) during two three-week rugby pre-season training blocks (mean ± SD).

Age (Years)	25 ± 2.7
Height (m)	1.87
Weight (kg)	105 ± 12.1

**Table 2:** Average Pittsburgh Sleep Quality and Epworth Sleepiness Scale Scores (Mean ± SD) in professional rugby union players (n = 25) during two three-week rugby pre-season training blocks.

	PSQI Score		ESS Score	
	Control	Intervention	Control	Intervention
Week 1	5.1 ± 2.2	4.9 ± 1.8	6.7 ± 3.5	7.0 ± 4.4
Week 2	4.9 ± 0.5	4.2 ± 1.7 ^	7.5 ± 2.7	4.8 ± 3.5 #
Week 3	5.4 ± 2.7	3.5 ± 1.0 ^	7.9 ± 3.6	6.0 ± 4.3

ESS > 10 = significant daytime sleepiness; PSQI > 5 = poor sleep quality. Magnitude indicates the change from week 1. # Small effect size; ^ Moderate effect size.

**Table 3:** Sleep variable changes (Mean ± SD) in professional rugby union players (n = 18) during two three-week rugby pre-season training blocks.

Sleep Variables	Control		Sleep Extension	
	Nights 1–8	Nights 9–18	Nights 1–6	Nights 11–17
Total time in bed (min)	516.0 ± 47.0	488.0 ± 41.0 #	529.3 ± 38.2	548.7 ± 44.8
Sleep latency (min)	17.6 ± 16.8	12.5 ± 11.5	13.0 ± 11.3	7.9 ± 4.9 #
Total sleep time (min)	391.0 ± 56.0	382.0 ± 36.0 #	422.4 ± 38.2	441.5 ± 44.1
Sleep efficiency (%)	75.0 ± 6.7	79.4 ± 4.8	79.9 ± 5.5	80.8 ± 5.4
Wake after sleep onset (WASO) (min)	103.0 ± 29.0	113.0 ± 85.0	93.5 ± 28.6	99.3 ± 28.6 #
Number of awakenings	28.0 ± 6.0	31.0 ± 16.0	28.2 ± 5.8	30.1 ± 4.6 #

Magnitude indicates the change between night block 1 and night block 2 within each treatment. # Small effect size.

**Table 4:** Biochemical changes (Mean ± SD) in professional rugby union players (n = 23) during two three-week rugby pre-season training blocks.

-	Control		Sleep Extension	
	Week1/Pre	Week3/Post	Week1/Pre	Week3/Post
α-Amylase (U·min^−1^)	163.1 ± 158.9	163.8 ± 155.0	163.5 ± 141.5	176.5 ± 179.7
IgA (μg·min^−1^)	31.0 ± 29.4	32.4 ± 27.5	26.2 ± 18.8	27.3 ± 22.7
Cortisol (nM)	14.3 ± 4.0	15.6 ± 5.1 #	6.0 ± 1.4	5.2 ± 1.7 #

Magnitude indicates the change from week 1 to week 3 within each month. # Small effect size.

**Table 5:** Reaction time changes (Mean ± SD) in professional rugby union players (n = 27) after sleep extension during a three-week rugby pre-season training block.

-	Pre	Post
Mean reaction time (ms)	228.8 ± 24.5	220.0 ± 18.4 #
Errors	2.6 ± 3.0	0.8 ± 1.1
Slowest 10% time (ms)	3.0 ± 0.5	3.3 ± 0.5 ^
Fastest 10% time (ms)	180.4 ± 11.0	175.4 ± 9.3 #

Magnitude indicates the change from start to end of sleep extension. # Small effect size; ^ Moderate effect size.
